# Chronic Elevation of Skeletal Muscle [Ca^2+^]_i_ Impairs Glucose Uptake. An *in Vivo* and *in Vitro* Study

**DOI:** 10.3389/fphys.2022.872624

**Published:** 2022-04-25

**Authors:** Arkady Uryash, Alfredo Mijares, Carlos E. Lopez, Jose A. Adams, Jose R. Lopez

**Affiliations:** ^1^ Division of Neonatology, Mount Sinai Medical Center, Miami Beach, FL, United States; ^2^ Centro de Biofísica y Bioquímica, Instituto Venezolano de Investigaciones Científicas, Caracas, Venezuela; ^3^ Department of Physiotherapy, Wellmax Medical Center, Miami, FL, United States; ^4^ Department of Research, Mount Sinai Medical Center, Miami Beach, FL, United States

**Keywords:** diabetes, calcium, dantrolene, GLUT4, skeletal muscle

## Abstract

Skeletal muscle is the primary site of insulin-mediated glucose uptake through the body and, therefore, an essential contributor to glucose homeostasis maintenance. We have recently provided evidence that chronic elevated intracellular Ca^2+^ concentration at rest [(Ca^2+^)_i_] compromises glucose homeostasis in malignant hyperthermia muscle cells. To further investigate how chronic elevated muscle [Ca^2+^]_i_ modifies insulin-mediated glucose homeostasis, we measured [Ca^2+^]_i_ and glucose uptake *in vivo* and *in vitro* in intact polarized muscle cells from glucose-intolerant *RYR1*-p.R163C and db/db mice. Glucose-intolerant *RYR1*-p.R163C and db/db mice have significantly elevated muscle [Ca^2+^]_i_ and reduced muscle glucose uptake compared to WT muscle cells. Dantrolene treatment (1.5 mg/kg IP injection for 2 weeks) caused a significant reduction in fasting blood glucose levels and muscle [Ca^2+^]_i_ and increased muscle glucose uptake compared to untreated *RYR1*-p.R163C and db/db mice. Furthermore, *RYR1*-p.R163C and db/db mice had abnormal basal insulin levels and response to glucose-stimulated insulin secretion. *In vitro* experiments conducted on single muscle fibers, dantrolene improved insulin-mediated glucose uptake in *RYR1*-p.R163C and db/db muscle fibers without affecting WT muscle fibers. In muscle cells with chronic elevated [Ca^2+^]_i_, GLUT4 expression was significantly lower, and the subcellular fraction (plasma membrane/cytoplasmic) was abnormal compared to WT. The results of this study suggest that i) Chronic elevated muscle [Ca^2+^]_i_ decreases insulin-stimulated glucose uptake and consequently causes hyperglycemia; ii) Reduced muscle [Ca^2+^]_i_ by dantrolene improves muscle glucose uptake and subsequent hyperglycemia; iii) The mechanism by which chronic high levels of [Ca^2+^]_i_ interfere with insulin action appears to involve the expression of GLUT4 and its subcellular fractionation.

## Introduction

Diabetes Mellitus (DM) is a severe chronic disease listed among the top 10 causes of death in adults. The global prevalence is estimated to be 578 million by 2030 and 700 million by 2045 (Saeedi et al., 2019). DM is characterized by chronic hyperglycemia resulting from muscle tissue’s inability to transport glucose, leading to eventual insulin resistance (Jia et al., 2016). Chronic hyperglycemia produced long-term and severe complications such as kidney failure, neuropathy, and cardiac dysfunction (Jia et al., 2018). More recently, diabetes has been reported to be a major contributor to mortality in patients with COVID-19 (Caballero et al., 2020).

Type 2 diabetes (T2D), by far the most common form of DM, affects people’s functional abilities and quality of life, leading to significant morbidity and premature mortality (Ramtahal et al., 2015). Despite significant advances and extensive research on the molecular basis of T2D, glycemic control remains suboptimal, and morbidity and mortality in diabetic patients remain high. Currently, treatment for patients with T2D includes reducing liver glucose metabolism (Wu et al., 2005), improving endogenous insulin secretion (Kaneko et al., 2019), limiting kidney glucose re-absorption (Vallon and Thomson, 2017), and lifestyle modification (diet and exercise) (Kolb and Martin, 2017). However, all of these therapies, except lifestyle changes, have limitations and drawbacks, and changing human behavior has proven to be very difficult. No pharmacologic treatment has been developed to target the pathogenic disturbances observed in skeletal muscle (muscle insulin resistance), although it is responsible for most of the glucose uptake of the entire body under insulin stimulation (Stump et al., 2006; Gogoi et al., 2014), and it is considered to be the hallmark of T2D (Lillioja et al., 1988; Shepherd and Kahn, 1999; DeFronzo, 2004).

Muscle insulin resistance reveals a suboptimal muscle glucose uptake response to normal insulin levels. Insights into the mechanisms that induce muscle insulin resistance are critical, but unfortunately, they are not fully understood. Among several possible alterations that contribute to insulin resistance in insulin target tissues is a chronic elevation of resting intracellular free Ca^2+^ concentration [(Ca^2+^)_i_] (Draznin et al., 1987; Draznin et al., 1989; Altamirano et al., 2019). An abnormal relationship between [Ca^2+^]_i_ and insulin resistance has been reported in skeletal muscle cells from patients susceptible to malignant hyperthermia (Altamirano et al., 2019), in adipocytes isolated from patients with T2D (Draznin et al., 1987; Byyny et al., 1992), and in muscle cells from patients with Duchenne muscular dystrophy (unpublished observations Lopez et al.). Furthermore, Park et al. (2009) and Lee et al. (1995) have shown that the expression of GLUT4 is inhibited by chronic elevation of [Ca^2+^]_i._ More recently, Tammineni et al. (2020) presented evidence that there are significant alterations in glucose metabolism in microsomal extracts of malignant hyperthermia muscle with elevated [Ca^2+^]_i_, evidenced by an increase in phosphorylated glycogen phosphorylase, glycogen synthase, and their Ca^2+^ dependent kinase.

We hypothesized that chronic elevated muscle [Ca^2+^]_i_ plays a critical role in muscle glucose intolerance observed in subjects with hyperglycemia. We support our postulate based on the fact that muscle cells of *RYR1*-p.163C (model of malignant hyperthermia) and db/db (T2D model) with chronic elevated [Ca^2+^]_i_ show reduced glucose uptake and hyperglycemia independent of genetic background. Reducing [Ca^2+^]_i_ with dantrolene, a muscle relaxant that lowers [Ca^2+^]_i_ in muscle and excitable cells (Lopez et al., 1987a; Lopez et al., 1992; Robin et al., 2017) improves skeletal muscle glucose uptake and hyperglycemia. Furthermore, muscle with impaired regulation of [Ca^2+^]_i_ and reduced glucose transport also shows alterations in the expression and subcellular distribution of the insulin-dependent glucose transporter GLUT4.

## Materials and Methods

### Experimental Models

Experiments were carried out in mice 3–4 months of age of both sexes and the same weight of *i)* C57BL/6, WT control; *ii) RYR1-*p.R163C, a mouse model of malignant hyperthermia (MH) that carried a mutation in the N-terminal domain of the isoform one of the ryanodine receptor (RyR1); *iii)* db/db mice, which have a deficiency in the leptin receptor that recapitulates most aspects of human diabetes disease. Experiments were conducted: *i) In vivo*, by measuring intracellular [Ca^2+^] in surgically exposed *gastrocnemius* fibers of anesthetized mice (100 mg/kg and xylazine 5 mg/kg) (Eltit et al., 2013); or *(ii) in vitro* using single muscle fibers obtained by enzymatic digestion of Flexor digitorum brevis muscle (FDB) muscles from anesthetized WT, *RYR1-p.R163C* and db/db mice, euthanized by cervical dislocation (Altamirano et al., 2014a). All animals were housed with the same number of mice per cage, under standard pathogen-free conditions at 23°C, with a regular 12:1-h light-dark cycle, provided standard mouse food and water *ad libitum* and kept in the Mount Sinai Medical Center vivarium.

### Glucose Determinations

Blood samples (5 µl) for glucose analysis were obtained from WT, *RYR1*-p.R163C, and db/db tail vein mice by direct flow or gently massaging; the glucose measurements were carried out using a handheld glucometer (AlphaTRAK^®^ glucose meter, Abbott Animal Health, Abbott Park, IL United States). For three consecutive tests, fastened mice with blood glucose levels >250 mg/dL were considered diabetic.

### Pharmacological Treatment With Dantrolene

WT, *RYR1*-p.R163C, and db/db mice were randomly assigned to control or dantrolene treatment. We studied six groups of mice: Group 1: WT mice were injected intraperitoneally (IP) with saline solution for 2 weeks. Group 2: WT mice were injected IP with dantrolene (1.5 mg/kg) for 2 weeks. We used a concentration of 1.5 mg/kg of dantrolene instead of the 2.5 mg/kg dose of MH treatment (Flewellen et al., 1983) because the former produces the desired effect on [Ca^2+^]_i_ without causing muscle weakness (Uryash et al., 2020). Group 3: *RYR1*-p.R163C mice were injected IP with saline solution for 2 weeks. Group 4: *RYR1*-p.R163C mice were injected IP with dantrolene (1.5 mg/kg) for 2 weeks; Group 5: db/db mice were injected IP with saline solution for 2 weeks. Group 6: db/db mice were injected intraperitoneally with dantrolene (1.5 mg/kg) for 2 weeks.

### Calcium Ion-Selective Microelectrodes

Double-barreled, Ca^2+^ selective microelectrodes were prepared from thin-walled 1.2 and 1.5 mm outside diameter (OD) borosilicate HCl-washed glass capillaries (PB150F-4, World Precision Instruments, FL, United States). The tip of the ion-selective barrel (1.5 mm OD) was silanized with dimethyldichlorosilane vapor and then filled with Ca^2+^ ionophore II (ETH 129, Sigma-Aldrich, MO, United States); the remainder of the barrel was filled with pCa7 (Eltit et al., 2013). The membrane potential barrel (1.2 mm OD) was filled with 3 M KCl. The double-barreled Ca^2+^ microelectrode was mounted on a modified plastic holder containing Ag/AgCl wires, which was attached to a head stage (input impedance >1011Ω), connected to a Duo 773 electrometer (World Precision Instruments, FL, United States). The Vm and Ca^2+^ specific potentials were acquired at a frequency of 1,000 Hz with the AxoGraph software (version 4.6; Axon Instruments, CA, United States) and stored on a computer for further analysis. Each Ca^2+^ selective microelectrode was individually calibrated before and after measurements, as previously described, and if the two calibration curves did not agree within 3 mV, the data from that microelectrode were discarded (Lopez et al., 1983; Eltit et al., 2013).

### Recording of [Ca^2+^]_i_ and Glucose Uptake in Muscle Fibers (*in Vivo*)

Anesthetized (100 mg/kg ketamine and 5 mg/kg xylazine) WT, *RYR1*-p.R163C, and db/db mice were placed on a heating plate (ATC1000, World Precision Instruments, FL, United States) to keep them euthermic (37°C). Hair was removed from the leg, and a small incision was made in the gastrocnemius muscle. The muscle fascia was partially removed, and the superficial muscle fibers were perfused at a rate of 3–5 ml/min with a warm Ringer to preserve moisture (37°C). Gastrocnemius muscle glucose uptake was evaluated by measuring the increase in fluorescence rate of the glucose analog {2- [N- (7-Nitrobenz-2oxa-1,3-diazol-4yl) amino] -2-deoxyglucose} (2-NBDG) (Osorio-Fuentealba et al., 2013), 30 min after IP injection of insulin 0.75 IU/kg body weight (Novo Nordisk Inc. NJ, United States). Muscle cells were incubated with 2-NBDG (100 µM) in a glucose-free Ringer for 30 min, washed several times, and then exposed to Ringer containing glucose. Muscle cells were excited at 467 nm, and the fluorescence emission was collected at 540 nm. Gastrocnemius 2-NBDG-loaded muscle cells were impaled with the double-barreled Ca^2+^selective microelectrode (Eltit et al., 2013), and measurements of muscle [Ca^2+^]_i_ and glucose uptake were carried out for each condition.

### Recording of Glucose Uptake in Single Muscle Fibers (*in Vitro*)

Experiments were conducted on FDB single muscle fibers from WT, *RYR1*-p.R163C, and db/db mice obtained by enzymatic digestion as described previously (Altamirano et al., 2014a). The following experiments were carried out 1) FDB single fibers were incubated with glucose-free Ringer-containing insulin (100 nM), exposed to 2-NBDG (100 µM) for 30 min, and then washed with glucose-free Ringer containing insulin for 15 min. The loaded 2-NBDG single fibers were exposed to normal Ringer-containing glucose, and fluorescence was recorded as previously described; 2) Single FDB fibers were incubated with glucose-free Ringer containing insulin (100 nM), supplemented with dantrolene (30 µM), exposed to 2-NBDG (100 µM) for 15 min, and washed for 15 min with glucose-free Ringer supplemented with dantrolene and insulin. The loaded 2-NBDG single fibers were exposed to Ringer-containing glucose plus dantrolene (30 µM), and fluorescence signals were recorded as previously described. Isolated muscles fibers were used for experimentation 4–6 h after isolation, and they were included in the study only if, at the end of the experiments, they responded vigorously to electrical stimulation (1 msec square pulse, 2.5 times threshold). All experiments were carried out in mammalian Ringer solution bubbled with 95% O_2_/5% CO_2_ at 37°C.

### Glucose Tolerance Test in Mice

To characterize muscle glucose impairment and hyperglycemia in *RYR1*-p.R163C and db/db mice, we carried out a glucose tolerance test (GTT) in the three overnight fasting experimental models described above. A glucose bolus (2 g/kg body weight) was delivered into the stomach by gavage stainless still needle -gauge 18- (Kent Scientific, CT, United States) between 8 and 10 a.m. to coincide with the hours of the normal physiological peak for glucose tolerance (Kalsbeek et al., 2010). Blood samples (5 µl) were taken from the tail vein immediately before (0 min) and 30-, 60-, 90-, and 120-min after glucose administration, and blood glucose concentration was measured using a handheld glucometer (AlphaTRAK^®^ glucose meter, Abbott Animal Health, Abbott Park, IL United States). The basal glucose value, the peak amplitude (30 min), and the glucose clearance were determined at 60-, 90-, and 120-min. The area under the curve (AUC) during the 120-min test duration was calculated using the trapezoid rule (GraphPad Prism 9.3.1 macOS, CA, United States). In a separate cohort of WT, *RYR1*-p.R163C, and db/db mice were divided into six groups (Pharmacological treatment with dantrolene) and the effect of dantrolene on GTT was discerned after the completion of dantrolene treatment as described above.

### Glucose-Stimulated Insulin Secretion in Mice

We examined glucose-stimulated insulin secretion in WT, *RYR1*-p.R163C, and db/db mice. Plasma insulin concentrations were measured using an insulin radioimmunoassay kit (Linco Research, St. Charles, Mo., USA). Mice fasted for 12 h were IP injected with glucose (2 g/kg body weight) and venous blood was collected at 0-, 30-, 60-, 90-, and 120-min. Glucose-stimulated insulin secretion was expressed as the AUC during the 120-min test duration using the same approach as described in the glucose tolerance test in mice.

### Western Blot and Fractionation of Subcellular Proteins

Another cohort of anesthetized mice (100 mg/kg ketamine and 5 mg/kg xylazine) WT, *RYR1*-p.R163C, db/db mice were injected with IP 0.75 IU/kg of insulin body weight (Novo Nordisk Inc. Novolin, United States) and 30 min later sacrificed by cervical dislocation. The gastrocnemius muscles were dissected, minced, and homogenized in a modified RIPA buffer (Altamirano et al., 2014b). Whole tissue homogenates were processed with a protein extraction kit (Sigma-Millipore, MA, United States), total protein concentrations were determined using the bicinchoninic acid (BCA) method (Thermo-Scientific, MA, United States), and aliquots were separated using PGE. After gel electrophoresis and protein transfer, the nitrocellulose membranes were incubated overnight at 4°C with primary antibodies specific for GLUT4 and actin (Abcam, MA, United States). Secondary fluorescent antibodies were used to produce protein bands for detection and quantification with a Storm 860 imaging system (GE Bio-Sciences, NJ, United States) and MyImageAnalysis software (Thermo-Fisher Scientific, MA, United States). The resulting signal data for the proteins of interest were normalized to actin values.

For subcellular protein fractionation, tissue samples were fractionated by detergent solubility with the Pierce subcellular protein fractionation kit (Thermo Fisher Scientific, MA, United States). 50 mg of tissue was washed gently with ice-cold phosphate-buffered saline and wiped to remove excess liquid. The remaining tissue was cut into small pieces and homogenized at 4°C with a protease inhibitor mix from the cytosol extraction buffer. Homogenized tissue was transferred to the Pierce tissue strainer with a 15 ml conical tube on ice. The strainers were centrifuged at 500 × *g* for 5 min and the supernatant (cytoplasmic extract) was transferred into a new tube on ice. The mixture of ice-cold MEB protease inhibitors was added to the pellet, vortexed in the tube for 5 s, incubated at 4°C for 10 min, and centrifuged at 3,000 × *g* for 5 min. The supernatant (membrane extract) was transferred into a new tube on ice. The fractions were kept on ice for immediate downstream applications and analysis or stored long-term at −80°C.

### Solutions

The mammalian Ringer solution had the following composition (in mM): 130 NaCl, 5 KCl, 1.8 CaCl_2_, 1 MgCl_2_, 5 glucose, and 10 HEPES, pH 7.4. Dantrolene (Sigma-Aldrich, MO, United States) solution was made by adding the desired concentration to the mammalian Ringer. The glucose-free Ringer solution had the same composition as the mammalian Ringer solution, but glucose was omitted, and mannitol was added to keep the osmolarity. The experimental solutions were changed using an automated perfusion system (Automate Scientific, CA, United States) and delivered through an unsharpened pipette positioned on top of the recorded muscle fiber. This arrangement allows us to make a rapid change with accurate timing.

### Statistics

Data are presented as mean ± standard deviation (SD). We excluded all data from muscle fibers that showed a resting membrane potential of less than −80 mV. We used the D’Agostino & Pearson test to determine whether the samples were normally distributed. We compared the experimental values using a one-way analysis of variance (ANOVA) and Tukey’s post-hoc test. A *p*-value < 0.05 was considered significant. A correlation analysis was performed to establish the relationship between two different variables (muscle [Ca^2+^]_i_ and muscle glucose uptake or muscle [Ca^2+^]_i_ and blood glucose levels). All statistical analyzes and the AUC were performed using GraphPad Prism 9.3.1 macOS (GraphPad Software, CA, United States).

## Results

### Abnormal Glucose Tolerance in RYR1-p.R163C and Db/Db Mice. Effects of Dantrolene

**FIGURE 1:**
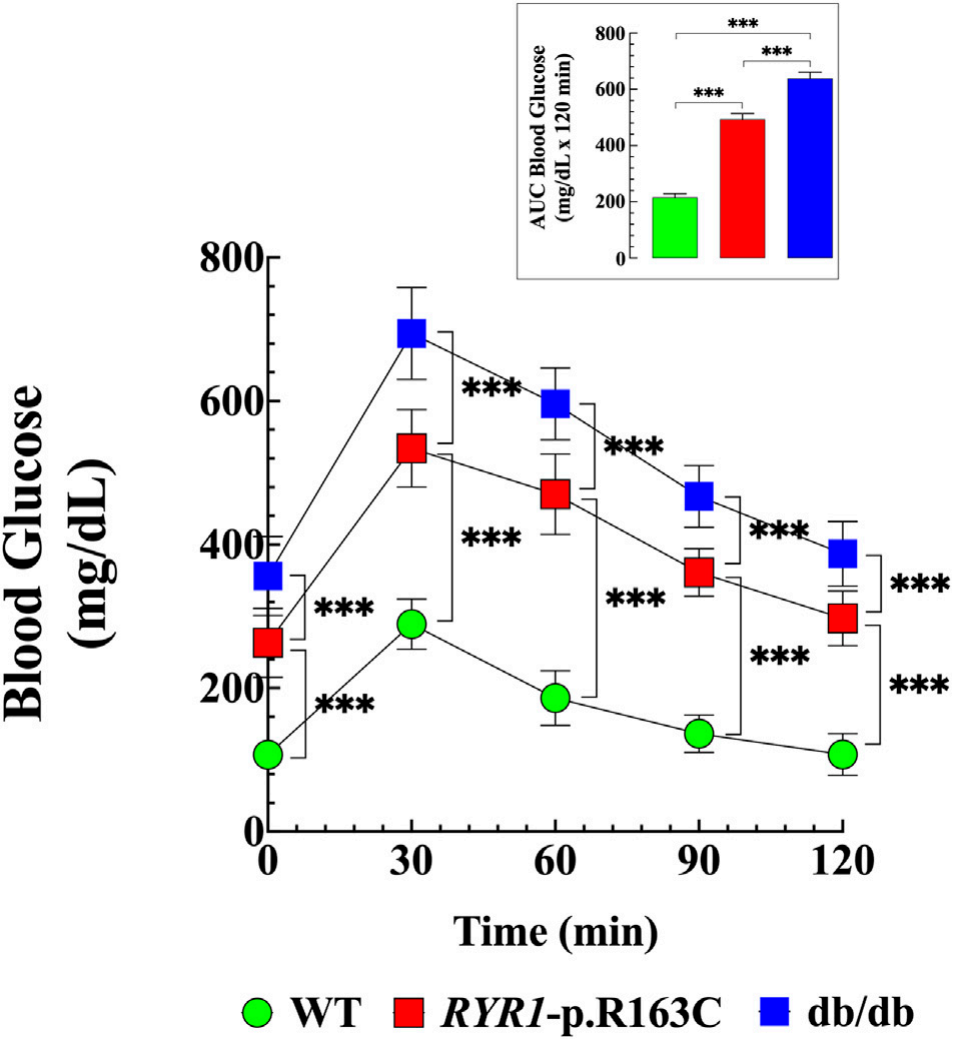
Abnormal glucose tolerance in RYR1-p.R163C and db/db. GTTs were carried out in fasting WT, *RYR1*-p.R163C, and db/db mice. Basal blood glucose levels were elevated in *RYR1*-p.R163C and db/db compared to WT mice. A notable difference in average peak amplitude at 30 min was found in *RYR1*-p.R163C and db/db compared to WT. The glucose tolerance was expressed as the AUC (Insert), which was calculated using the trapezoid rule over the 120-min test duration (GraphPad Prism software 9.0). Values are expressed as mean ± S. D; experiments were conducted in *n*_*mice*_ *=* 6 per genotype. Statistical analysis was performed using a one-way analysis of variance (ANOVA) followed by Tukey’s multiple comparisons. *** = *p* < 0.001.

We have reported altered glucose homeostasis in patients and rodents with aberrant skeletal muscle [Ca^2+^]_i_ (Altamirano et al., 2019). To further characterize glucose impairment in aberrant [Ca^2+^]_i_ mice, glucose tolerance tests (GTT) were performed in fasted (12 h) WT, *RYR1-p.R163C,* and db/db mice. Figure 1 shows the summary data of the GTTs and the area under the curve (Figure 1 insert) throughout the study (120-min). There was a significantly higher value for fasting blood glucose levels in *RYR1*-pR163C (263 ± 48 mg/dL, *p* < 0.001) and in db/db (356 ± 55 mg/dL, *p* < 0.001) compared to WT (107 ± 15 mg/dL). Furthermore, there was a robust difference in average peak amplitude at 30-min (1.8 times in *RYR*1-pR163C and 2.4 times in db/db greater than WT values) (Figure 1). Furthermore, *RYR*1-pR163C and db/db mice had slower glucose clearance than WT mice at 60-, 90-, and 120-min and showed a significantly greater area under the curve than WT mice (2.3 times and 3 times, respectively, *p* < 0.001) (Figure 1 insert).

**FIGURE 2:**
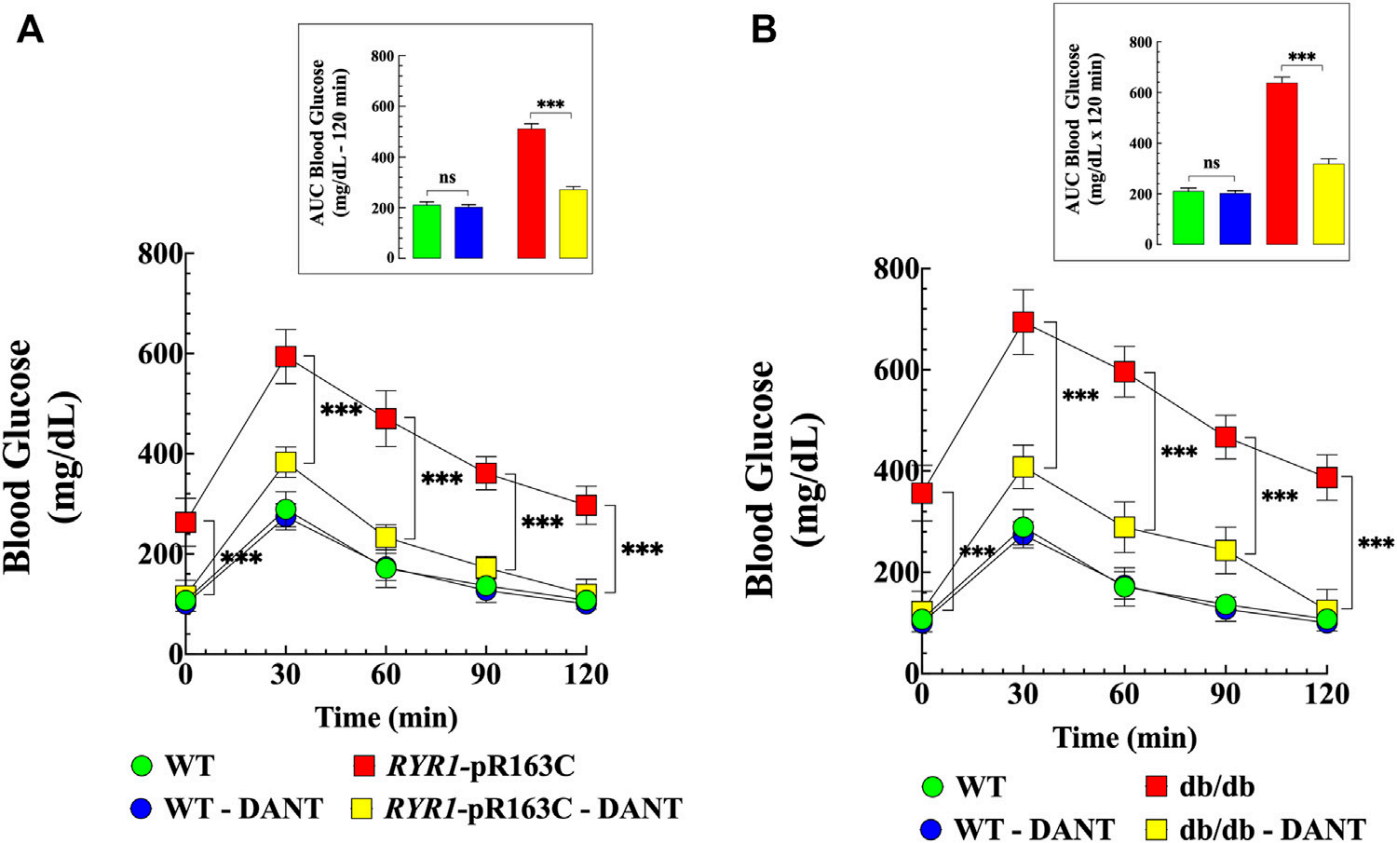
Dantrolene improves glucose tolerance in RYR1-p.R163C and db/db mice. GTT was carried out before and after dantrolene treatment (1.5 mg/kg intraperitoneally daily for 2 weeks) in WT, *RYR1*-p.R163C, and db/db mice. Dantrolene significantly reduced fasting glycemia in *RYR1*-p.R163C **(A)** and db/db mice **(B)** and improved response to glucose load at all times studied in *RyR1*-p.R163C and db/db compared to untreated mice. The insets in Figures **(A, B)** represent the area under the curve before and after dantrolene treatment for 120 min. The results represent the mean ± S. D from *n*_*mice*_ *=* 5 per genotype. Statistical analysis was performed as described above. ns = non significative; *** = *p* < 0.001.

To further elucidate the role of muscle [Ca^2+^]_i_ in glucose homeostasis, WT, *RYR1*-pR163C, and db/db mice were treated with dantrolene (1.5 mg/kg IP, for 2 weeks), which significantly reduces [Ca^2+^]_i_ (Lopez et al. unpublished results). Mice were divided into 6 groups as previously described (Pharmacological treatment with dantrolene), and glucose tolerance tests were performed at the end of the pharmacological treatment. Dantrolene normalized blood glucose levels in *RYR1*-pR163C (2.2-times, *p* < 0.001) and db/db mice (2.9-times, *p* < 0.001), reduced the maximum amplitude 30 min after glucose load by 1.5 times (*p* < 0.001) in *RYR1*-pR163C and by 1.7 times (*p* < 0.001) in db/db mice (Figures 2A,B). Furthermore, dantrolene accelerated glucose clearance at 60-, 90-, and 120-min, significantly reducing the area under the curve in *RYR1*-pR163C mice by 1.9 times (*p* < 0.001) and in db/db mice by 2 times (*p* < 0.001) compared to untreated mice (Figures 2A,B inserts). Dantrolene did not significantly reduce blood glucose levels or the area under the curve in WT (Figures 2A,B and insert).

### Abnormal Glucose-Stimulated Insulin Secretion

**FIGURE 3:**
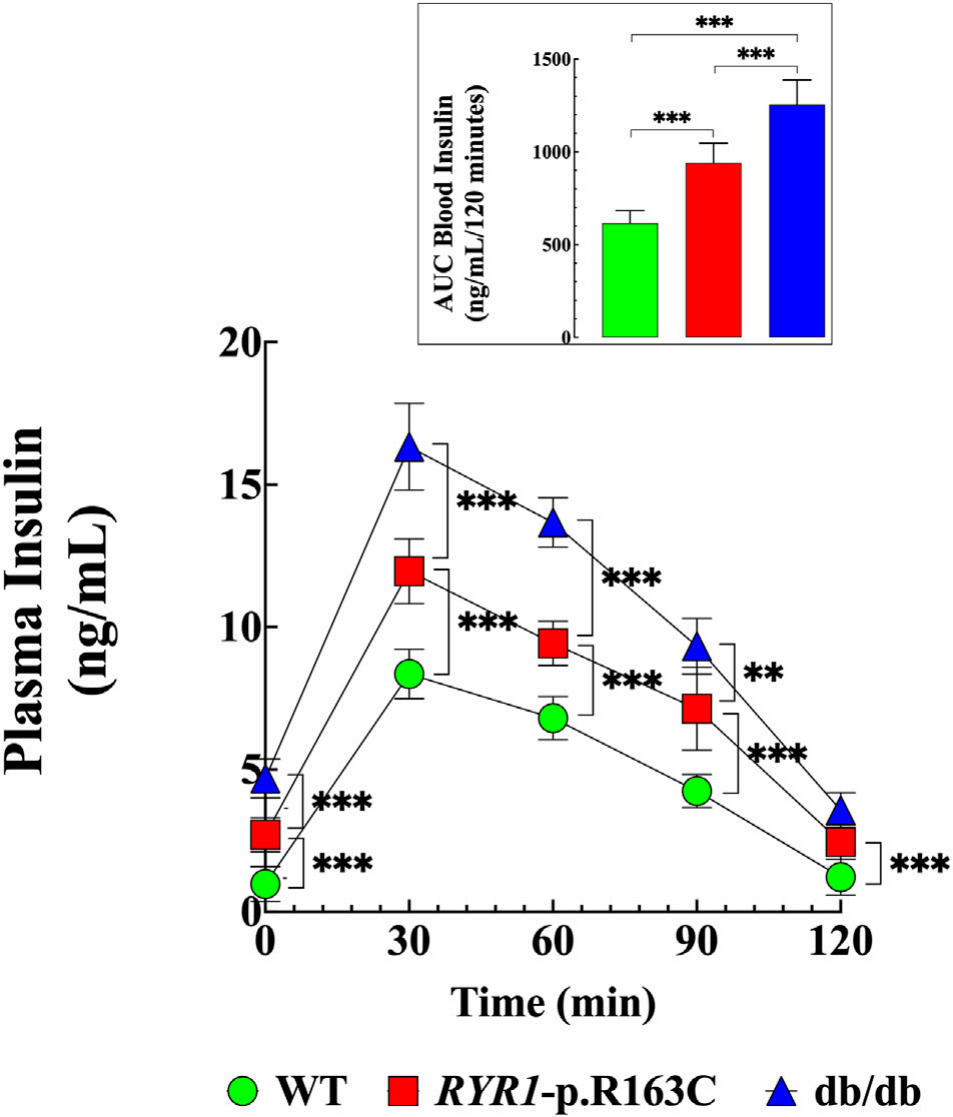
Abnormal insulin levels in response to glucose challenge. Insulin levels were determined in WT, *RYR1-*p.R163C, and db/db mice after a glucose load. The basal level of insulin was higher in *RyR1*-p.R163C and db/db than in WT mice and during the test duration of 120 min. The insert shows the AUC calculated using the trapezoid rule of the insulin determination (GraphPad Prism software 9.0). Values are expressed as mean ± S.D. Experiments were conducted in *n*_*mice*_ *=* 6 per genotype. Statistical analysis was performed as described above. ** = *p* < 0.01, *** = *p* < 0.001.

Insulin is a hormone essential for the regulation of blood glucose levels, and elevated concentration is a cardinal feature of T2D (Warram et al., 1990). We analyzed plasma insulin levels in response to glucose stimulation in WT, *RYR1*-p.R163C, and db/db mice. Mice fasted for 12 h were injected IP with glucose (2 g/kg body weight), and tail venous blood was obtained at 0-, 30-, 60-, 90- and 120-min after injection. Fasting insulin levels were 2.7 times (*p* < 0.001) higher in *RYR1*-pR163C and 4.7 times (*p* < 0.001) in db/db than in WT (Figure 3). The average peak amplitude (30 min) after glucose load was 1.4-times higher in *RYR*1-pR163C (*p* < 0.001) and 2 times in db/db (*p* < 0.001) compared to WT mice (Figure 3). Additionally, the decay of blood insulin levels was slower (60-, 90-, and 120-min) in *RYR1*-pR163C and db/db mice compared to WT. Furthermore, the area under the curve (0–120 min) was 1.5 times greater in *RYR1*-pR163C (*p* < 0.001) and 2 times in db/db (*p* < 0.001) compared to WT (Figure 3 insert).

### Effects of Dantrolene on Resting [Ca^2+^]_i_ in RYR1-p.R163C and Db/Db Skeletal Muscle Fibers

**FIGURE 4:**
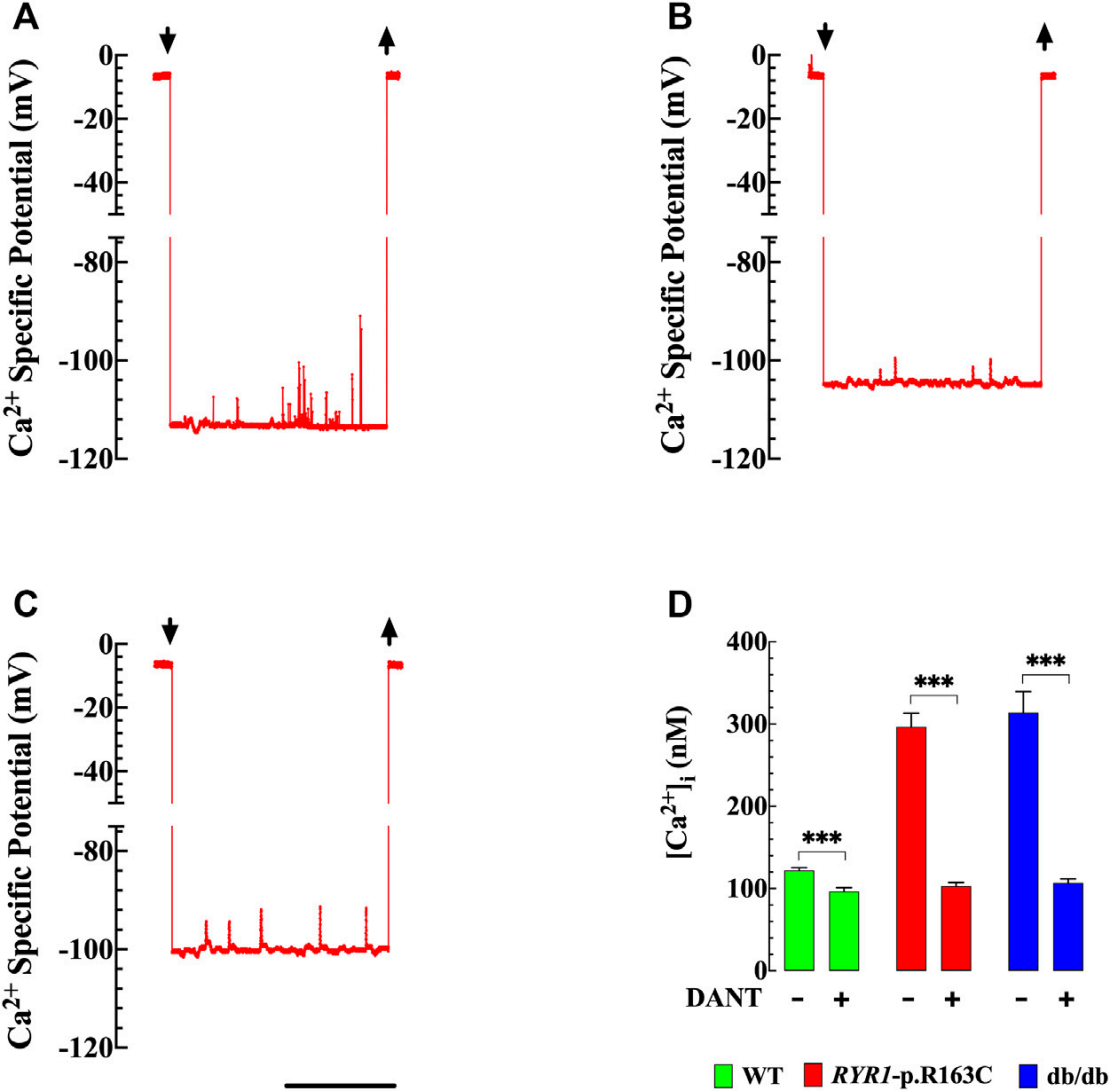
[*Ca* ^*2+*^]_*i*_ in intact skeletal muscle cells. Effect of dantrolene. The records represent the specific potential of Ca^2+^ obtained from WT, *RYR1*-pR163C, and db/db muscle cells. [Ca^2+^]_i_ was calculated using the corresponding microelectrode calibration curve (Lopez et al., 1983). **(A)** Shows a representative record of the Ca^2+^ specific potential obtained from a WT muscle cell, [Ca^2+^]_i_ was 119 nM. The upper left and right arrows indicate Ca^2+^ microelectrode impalement and withdrawal, respectively; **(B)** Ca^2+^ specific potential recorded from *RYR1*-pR163C muscle fiber, [Ca^2+^]_i_ was 284 nM; **(C)** Ca^2+^ specific potential recorded from a db/db muscle fiber; [Ca^2+^]_i_ was 329 nM. **(D)** [Ca^2+^]_i_ was significantly higher in *RYR1*-pR163C and db/db than WT muscle cells. Dantrolene treatment (1.5 mg/kg IP daily for 2 weeks) reduced [Ca^2+^]_i_ in all genotypes; however, the reduction was greater in *RYR1 p*. R163C (2.8 times) and db/db (2.9 times) than in WT muscles (1.2 times). Values are expressed as mean ± S. D; experiments were carried out in *n*_*mice*_ *=* 6 per genotype; *n*_*cells*_ *=* 13–16/genotype group. Statistical analysis was performed as described above. *** = *p* < 0.001. Calibration bar = 1 min.

Abnormal [Ca^2+^]_i_ is a common underlying phenomenon in the pathophysiology of various skeletal muscle diseases (Lopez et al., 1987b; Lopez et al., 1995; Lopez et al., 2005; Lopez et al., 2008). Muscle [Ca^2+^]_i_ was measured *in vivo* in superficial gastrocnemius fibers in anesthetized WT, *RYR1*-p.R163C, and db/db using Ca^2+^ selective microelectrodes. We confirmed in *RYR1*-p.R163C and found in db/db that [Ca^2+^]_i_ in skeletal muscle is higher than in WT. Figures 4A–C show typical records of [Ca^2+^]_i_ measured in intact, polarized skeletal muscle fibers of WT (A) *RYR1*-p.R163C (B) and db/db (C), respectively. The average values of [Ca^2+^]_i_ in WT was 122 ± 3 nM (*n* = 16), while *RYR1*-p.R163C was significantly more elevated 297 ± 17 nM (*n* = 14, *p* < 0.001 compared to WT) and in db/db 314 ± 25 nM (*n* = 13, *p* < 0.001 compared to WT) (Figure 4D).

Pretreatment of WT, *RYR1-p.R163C*and db/db mice with dantrolene (1.5 mg/kg intraperitoneally, for 2 weeks), caused a significant reduction in skeletal muscle [Ca^2+^]_i_ in all mice. In WT [Ca^2+^]_i_ was reduced by 1.3 times (96 ± 5 nM, *n* = 13, *p* < 0.001), in *RYR1*-p.R163C by 2.8 times (103 ± 4 nM, *n* = 13, *p* < 0.001) and db/db by 2.9 times (107 ± 5 nM, *n* = 14, *p* < 0.001) (Figure 4D).

### Chronic Increases in [Ca^2+^]_i_ Reduce Glucose Uptake in Skeletal Muscle. Effects of Dantrolene

**FIGURE 5:**
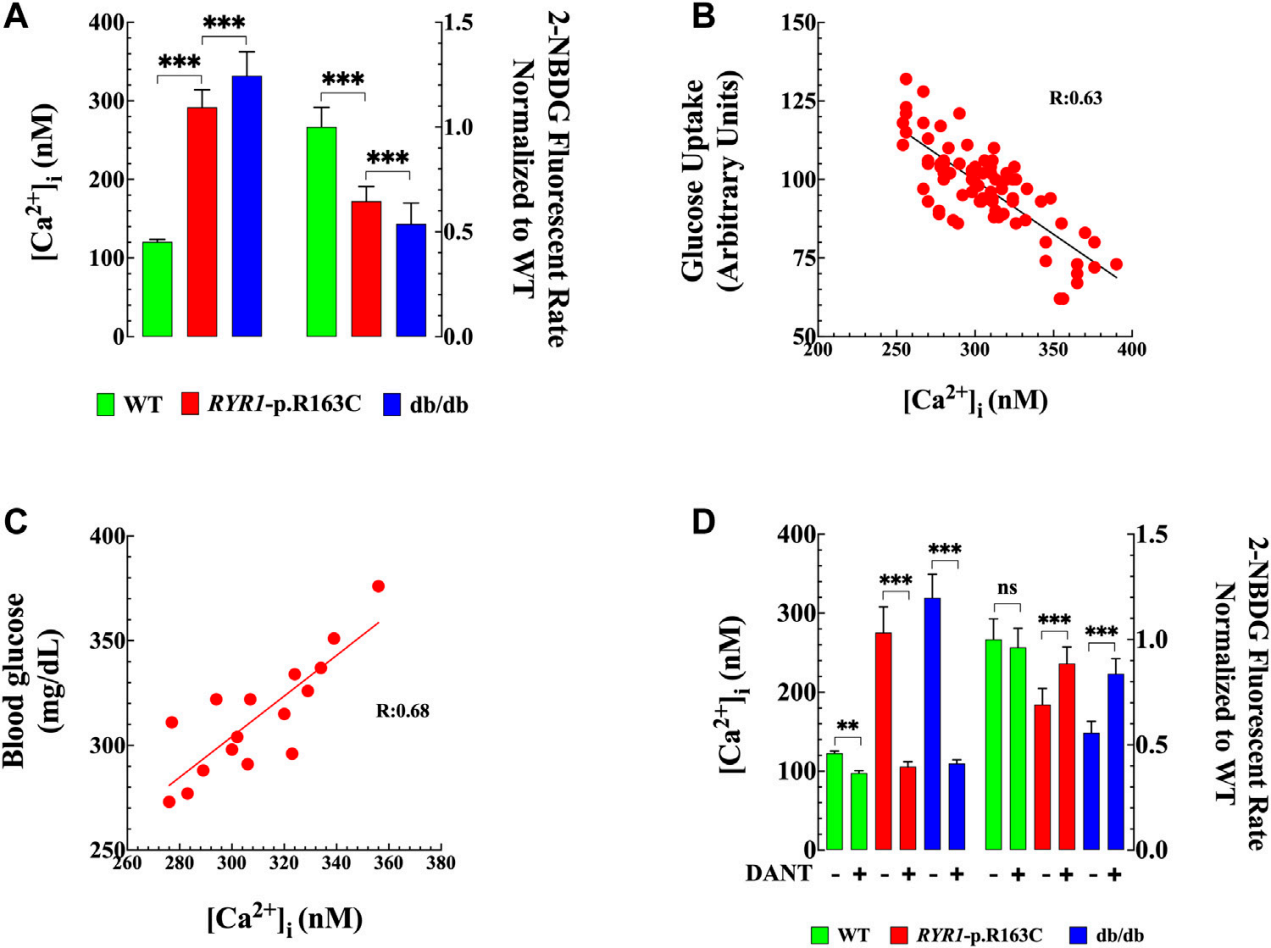
Increased [Ca^2+^]i impairs glucose uptake in muscle fibers. [Ca^2+^]_i_ and glucose uptake were recorded simultaneously *in vivo* in intact gastrocnemius fibers WT and *RyR1*-p.R163C and db/db using double-barreled selective Ca^2+^ microelectrodes and the 2-NBDG fluorescent glucose analog. **(A)** Left panel: Intracellular [Ca^2+^] was higher in *RYR1*-p.R163C and db/db than in the WT muscle. Right panel: Glucose uptake was significantly reduced in *RYR1*-p.R163C and db/db compared to WT muscle. All fluorescence signals were normalized to WT. **(B)** A positive correlation between muscle glucose uptake and [Ca^2+^]_i_ was found in *RyR1*-p.R163C and db/db (R = 0.63). An increase in muscle [Ca^2+^]_i_ caused a decrease in glucose uptake. **(C)** Show a positive correlation analysis between mice’s blood glucose (mg/dL) and muscle [Ca^2+^]_i_ (nM) (R = 0.68). Increased muscle [Ca^2+^]_i_ caused a more elevated blood glucose concentration. **(D)** Left panel: [Ca^2+^]_i_ was elevated in *RYR1*-p.R163C and db/db compared to WT muscle. Dantrolene treatment significantly reduced [Ca^2+^]_i_ and all genotypes. Right panel: insulin-dependent glucose uptake was reduced in *RYR1*-p.R163C and db/db compared to WT muscle. Dantrolene improved glucose uptake in the *RYR1-*p.R163C and db/db muscles, with no significant effect in WT. All fluorescence signals were normalized to WT. The results represent the mean ± S. D from *n*_*cells*_ *=* 14–52/genotype group*,* isolated from *n*_*mice*_ *=* 5–6 per genotype; *n*_*cells*_ *=* 14–52/genotype group. Statistical analysis was performed as described above. ** = *p* < 0.01; *** = *p* < 0.001.

To establish the direct correlation between resting muscle [Ca^2+^]_i_ and glucose uptake, in a different set of experiments, we simultaneously measured [Ca^2+^]_i_ and glucose uptake *in vivo* in superficial intact gastrocnemius fibers of anesthetized WT, *RYR1*-p.R163C, and db/db [Ca^2+^]_i_ was measured using double-barreled Ca^2+^ selective microelectrodes (Eltit et al., 2013), and glucose uptake was measured with the 2-NBDG fluorescent glucose analog (Zou et al., 2005). [Ca^2+^]_i_ in *RYR1*-p.R163C was 2.4 times more elevated (292 ± 22 nM, *n* = 40, *p* < 0.001 compared to WT) and in db/db was 2.7-times (332 ± 31 nM, *n* = 31, *p* < 0.001 compared to WT) (Figure 5A left y-axis). On the other hand, a significant reduction in insulin-dependent glucose uptake was observed in *RYR1*-p.R163C (1.5 times, *p* < 0.001) and in db/db (*p* < 0.001, 1.8 times) compared to WT (Figure 5A right y-axis). Figure 5B shows a negative linear relationship between muscle [Ca^2+^]_i_ and glucose uptake (R = 0.63), and Figure 5C shows a positive linear relationship between muscle [Ca^2+^]_i_ (average per mouse) and the glucose blood level of *RYR1*-p.R163C and db/db mice (R = 0.68). In summary, mice with impaired muscle [Ca^2+^]_i_ handling have significantly reduced glucose uptake and subsequent hyperglycemia*.*

To determine the impact of muscle reduction [Ca^2+^]_i_ on insulin-dependent glucose uptake, in another set of experiments, we simultaneously measured [Ca^2+^]_i_ and 2-NBDG fluorescence *in vivo* in the superficial fibers of the gastrocnemius muscle from WT, *RYR1-*p.R163C, and db/db mice treated with dantrolene (1.5 mg/kg intraperitoneally for 2 weeks). Dantrolene significantly reduced [Ca^2+^]_i_ by 1.2 times in WT (*p* < 0.01 compared to untreated WT muscles), 2.6 times in *RYR1*-p.R163C (*p* < 0.001 compared to untreated *RYR1*-p.R163C muscles) and 2.9 times in db/db muscle cells (*p* < 0.001 compared to untreated db/db muscles). Furthermore, dantrolene improved glucose uptake by 1.3 times in *RYR1*-p.R163C (*p* < 0.001 compared to untreated *RYR1*-p.R163C muscles) and 1.5 times in db/db (*p* < 0.001 compared to untreated db/db muscles) (Figure 5D). Dantrolene did not modify glucose uptake in WT (*p* = 0.722) (Figure 5D).

### Effect of Dantrolene on Glucose Uptake in Single Muscle Fibers

**FIGURE 6:**
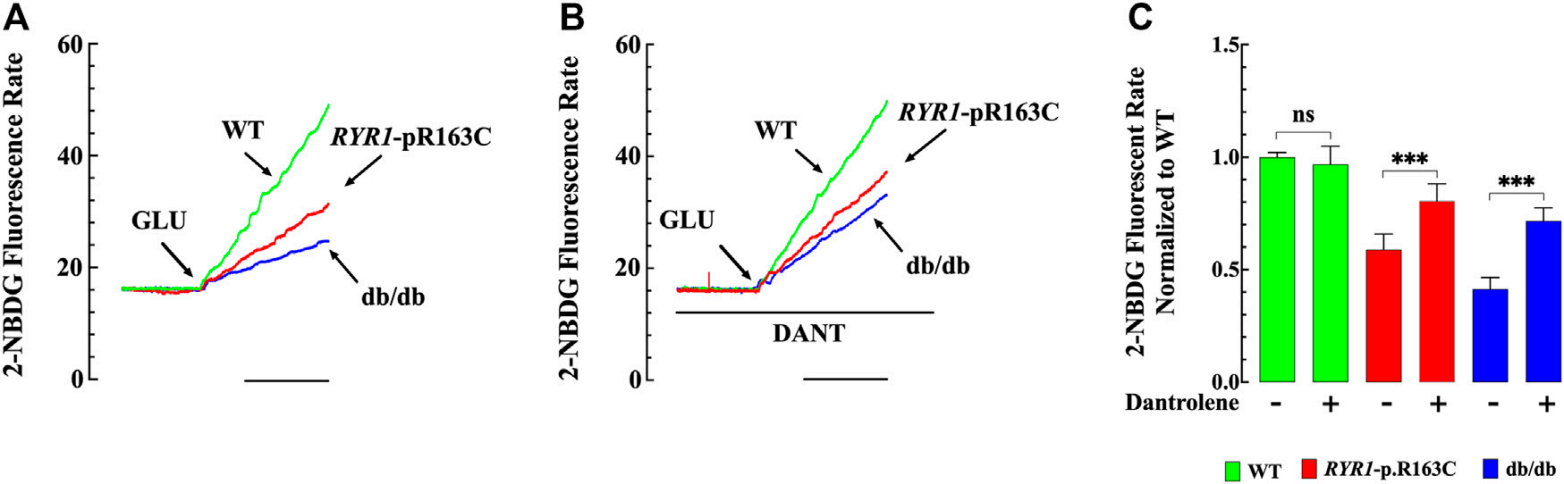
Glucose uptake in WT, *RYR1*-p.R163C, and db/db single fibers. Effects of dantrolene. **(A)** Single muscle fiber recordings of 2-NBDG fluorescent signals from WT, *RYR1-*p.R163C, and db/db muscle. **(B)** Typical records of NBDG fluorescent signals show the effect of dantrolene on insulin-dependent glucose uptake in single muscle fibers WT, *RYR1*-p.R163C, and db/db. **(C)** Shows the summary of abnormal insulin-dependent glucose uptake in *RYR1-*p.R163C and db/db single muscle fibers and the effects of dantrolene. The florescent rate values were normalized to untreated WT and expressed as mean ± S. D; experiments were carried out in *n*_*mice*_ *=* 5 per genotype; *n*_*fibers*_ *=* 9–14/genotype group. Statistical analysis was performed as described above. *** = *p* < 0.001. Calibration bar = 5 min.

Insulin resistance to the skeletal muscle is one of the first detectable defects in T2D. We evaluated insulin-dependent glucose uptake in single fibers loaded with 2-NBDG from WT, *RYR1*-p.R163C, and db/db mice. The 2-NBDG fluorescent rate was significantly reduced in *RYR1*-p.R163C (*p* < 0.001) and db/db (*p* < 0.001) compared to WT (Figure 6A, C).

In another set of experiments, we determined whether a reduction in [Ca^2+^]_i_ caused by dantrolene was relevant to its ability to improve insulin resistance. Experiments were carried out in 2-NBDG loaded FDB single fibers from WT, *RYR1*-p.R163C, and db/db mice pretreated with dantrolene (20 μM) for 15 min. We selected dantrolene (20 μM) because such a concentration significantly reduced [Ca^2+^]_i_ without compromising glucose transport in muscle cells (Lopez et al. unpublished experiments). Pretreatment with dantrolene caused a significant increase in the 2-NBDG rate by 1.4 times in *RYR1*-p.R163C (*p* < 0.001) and 1.7 times in db/db (*p* < 0.001) compared with untreated *RYR1*-p.R163C and db/db muscle fibers respectively (Figure 6B,C). Dantrolene did not change insulin-dependent glucose uptake in WT (*p* = 0.877 compared to untreated) (Figures 6B,C).

### GLUT4 Expression and Subcellular Proteins Fractionation in Muscle With Abnormal [Ca^2+^]_i_

**FIGURE 7:**
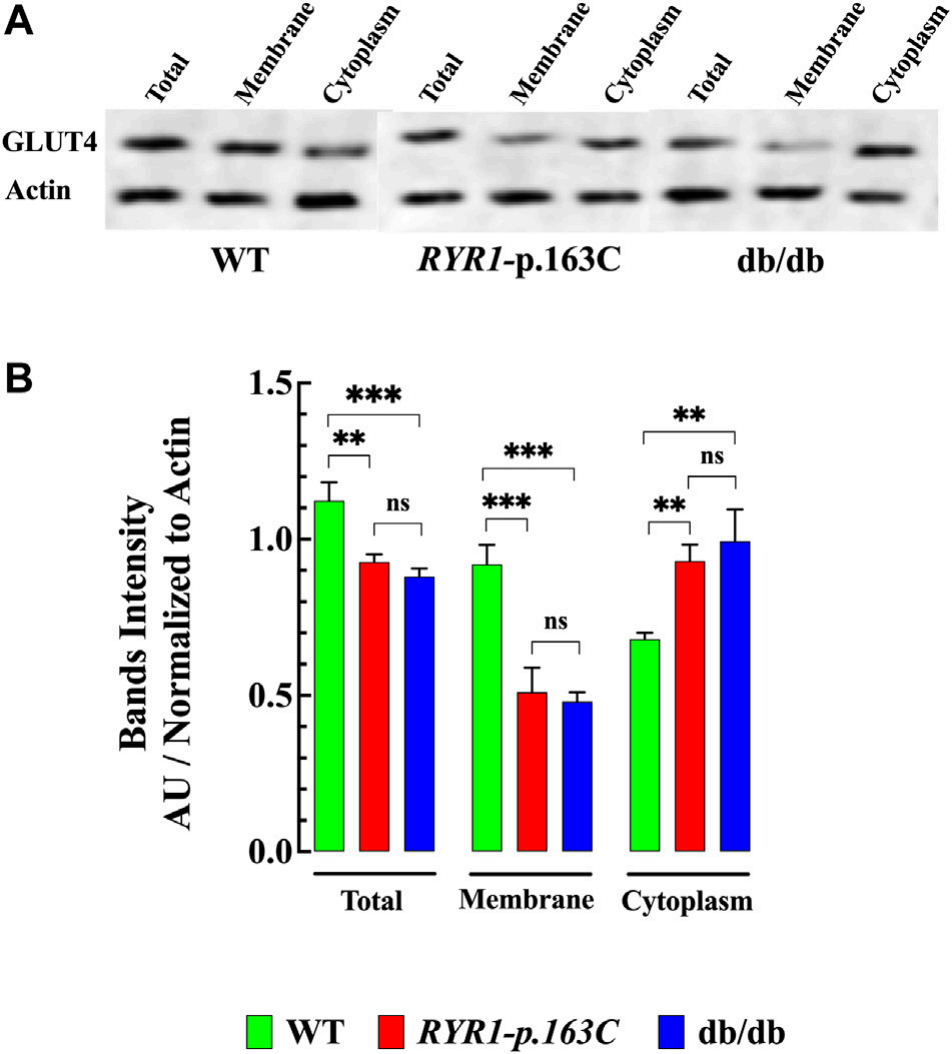
Expression and subcellular distribution of GLUT4 in skeletal muscle. The total expression of GLUT4 was significantly lower in *RYR1*-pR163C and db/db *gastrocnemius* muscle compared to age-matched WT muscle. Furthermore, the GLUT4 subcellular membrane fraction was significantly reduced in *RYR1*-pR163C and db/db muscles compared to WT; in contrast, the cytoplasmic fraction of GLUT4 was higher in *RYR1*-pR163C and db/db muscles than in WT. **(A)** Representative immunoblots collected from WT, *RyR1*-p.R163C, and the db/db muscle homogenates. **(B)** Densitometric quantification of the global and subcellular distribution of GLUT4 in WT, *RYR1*-pR163C, and db/db muscle. Values are expressed as mean ± S.D. Experiments were carried out in *n*_*mice*_ *=* 3 per genotype, *n*_*WB*_ = 5. Statistical analysis was performed as described above. ns = non significative, ** = *p* < 0.01, *** = *p* < 0.001.

Next, we determine whether elevated [Ca^2+^]_i_ interferes with the expression and subcellular distribution of GLUT4, an insulin-regulated glucose transporter, in homogenates of the *RYR1*-p.R163C and db/db muscle compared to WT. Our data show that GLUT4 expression was significantly lower in *RYR1*-pR163C (1.2-times, *p* < 0.05) and in the db/db muscle (1.3-times, *p* < 0.01) compared to the WT muscle (Figure 7A,B **)**. Furthermore, the subcellular fractionation of GLUT4 attached to the plasma membrane was smaller in the muscle *RYR1*-pR163C (1.8-times, *p* < 0.001) and db/db (1.9-times, *p* < 0.001) muscle compared to the WT muscle, while the cytoplasmic fraction was greater in *RYR1*-pR163C (1.4-times, *p* < 0.01) and db/db (1.5-times, *p* < 0.01) muscle compared to the WT muscle (Figures 7A,B).

## Discussion

This study represents the first report that combines the determination of [Ca^2+^]_i_ using Ca^2+^ selective microelectrodes and a fluorescent glucose analog (2-NBDG) in intact skeletal muscle (*in vivo*), allowing the assessment of whether elevating [Ca^2+^]_i_ causes insulin resistance in muscle cells in two experimental models with different genetic backgrounds. The more important finding of the present study are:

### In Vivo

*i)* Muscle cells from glucose-intolerant *RYR1*-p.R163C and db/db mice show a chronic elevated [Ca^2+^]_i_ and a reduced glucose uptake mediated by insulin. Dantrolene reduces muscle [Ca^2+^]_i_ and cause an increase in muscle glucose uptake. Furthermore, dantrolene decreases fasting blood glucose levels and improves the glucose tolerance test in *RYR1*-p.R163C and db/db mice.
*ii) RYR1*-p.R163C and db/db mice have an abnormal blood insulin level and an anomalous response to glucose-stimulated plasma insulin.
*iii)* There is an optimal muscle [Ca^2+^]_i_ (100–130 nM) that facilitates muscle insulin glucose uptake. However, chronic elevation of [Ca^2+^]_i_ outside of this optimal concentration inhibits insulin-dependent glucose uptake.

### In Vitro

*i)* Dantrolene improves insulin-mediated glucose uptake in *RYR1*-p.R163C and db/db muscle fibers, with no effect on WT.
*ii)* The expression and translocation of GLUT4 from intracellular vesicle to the plasma membrane was altered in both experimental models with chronically elevated [Ca^2+^]_i_. Muscle GLUT4 expression was significantly lower and subcellular protein fractionation was altered in *RYR1*-p.R163C and db/db mice. The GLUT4 fractionation attached to the plasma membrane was lower in the muscle *RYR1*-pR163C and db/db, while the cytoplasmic fraction of *RYR1*-pR163C and db/db was higher compared to the WT muscle.

### Abnormal Muscle [Ca^2+^]_i_, Glucose Uptake, and Insulin Resistance

The skeletal muscle is the largest sink site for insulin-dependent glucose uptake. Alterations in glucose homeostasis cause pathophysiological conditions such as T2D, the leading cause of millions of deaths worldwide every year. Alteration in insulin sensitivity, called insulin resistance, is a specific and early characteristic of T2D that results in impaired glucose removal (DeFronzo and Tripathy, 2009; Petersen and Shulman, 2018).

In the present investigation, we have found that increased [Ca^2+^]_i_ reduce muscle cells’ sensitivity to insulin. [Ca^2+^]_i_ in skeletal muscle is tightly regulated in terms of spatial and temporal distributions by various intracellular regulatory mechanisms, balancing Ca^2+^ influx and release from intracellular organelles with intracellular sequestration and extracellular extrusion (Cali et al., 2017). Therefore, muscle [Ca^2+^]_i_ is preserved at a low level (100–120 nM) in quiescent muscle cells against a bulky extracellular concentration gradient (∼2 mM) (Lopez et al., 1983; Lopez et al., 1988). The abnormalities in [Ca^2+^]_i_ observed in *RYR1*-p.163C and db/db are consistent with previous reports in malignant hyperthermia susceptible muscle cells (Lopez et al., 1985; Yang et al., 2006; Eltit et al., 2010; Altamirano et al., 2014a) and from experimental models and patients with diabetes (Draznin et al., 1989; Ohara et al., 1991; Tschope et al., 1991). Alterations in the plasma membrane Ca^2+^ ATPase, the sarcoplasmic reticulum Ca^2+^ ATPase, and the Na ^+^-Ca^2+^ exchange have been reported (Condrescu et al., 1987; Makino et al., 1987; Mickelson and Louis, 1996; Altamirano et al., 2014a; Eshima, 2021) in malignant hyperthermia and diabetic muscles. Furthermore, a leak of Ca^2+^ from the type 1 ryanodine receptor (RyR1) channels (Yang et al., 2007) and a high influx of transmembrane Ca^2+^ through TRPC channels have been described (Eltit et al., 2010; Rafael Lopez et al., 2020) malignant hyperthermia muscle. Therefore, alterations in these intracellular Ca^2+^ regulatory mechanisms can contribute to the impairment of [Ca^2+^]_i_ observed in RYR1-p.163C and db/db muscle cells.

In the insulin signaling cascade that promotes glucose uptake in skeletal muscle, intracellular Ca^2+^ seems to play two distinct roles: *i)* permissive and *ii)* dynamic role. A permissive role in which [Ca^2+^]_i_ modulates the muscle response to insulin (facilitating or inhibiting it). A dynamic role where a transient and local subsarcolemmal elevation of intracellular [Ca^2+^] is required for muscle insulin-dependent glucose uptake (Bruton et al., 1999; Lanner et al., 2006). Insulin-dependent elevation of intracellular [Ca^2+^] appears to be mediated by the canonical transient receptor potential (TRPC) 3 and 6, as GSK2332255B, a selective TRPC3/6 antagonist (Washburn et al., 2013), prevented the increase in [Ca^2+^]_i_ associated with insulin effects in human myotubes (Uryash et al. unpublished results).

Under physiological conditions, insulin stimulates glucose uptake in skeletal muscle, cardiac muscle, and adipose tissue to maintain glucose homeostasis (Jaiswal et al., 2019). We have shown that chronic increase in muscle [Ca^2+^]_i_ caused insulin resistance in two mouse models, *RYR1-*p.163C, a model of malignant hyperthermia (Yang et al., 2006), and db/db, a model of T2D (Coleman, 1982; Leibel et al., 1997). Similarly, an association between elevated [Ca^2+^]_i_ and insulin resistance has been reported in human fat cells and rats (Draznin et al., 1988; Draznin et al., 1989; Reusch et al., 1991) and muscle in which [Ca^2+^]_i_ was elevated pharmacologically (Westfall and Sayeed, 1990). Insulin resistance has also been reported in the aging process (Defronzo, 1979), and various pathologies such as heart failure (Swan et al., 1997), Duchenne muscular dystrophy (Freidenberg and Olefsky, 1985), uremic patients (Siew and Ikizler, 2010), Alzheimer’s disease (Dominguez et al., 2014) and MH (Altamirano et al., 2019). Interestingly, most of these pathologies share a common disorder: intracellular Ca^2+^ dysfunction reflected in elevated [Ca^2+^]_i_ (Lopez et al., 1987b; Lopez et al., 2005; Lopez et al., 2008; Altamirano et al., 2019; Uryash et al., 2020).

*RYR1-*p.163C and db/db mice had elevated circulating insulin levels and abnormal peak amplitude after glucose load compared to WT. Hyperinsulinemia is a compensatory increase in pancreatic insulin production (Petersen and Shulman, 2018) caused by skeletal muscle insulin resistance (DeFronzo and Tripathy, 2009). The hyperinsulinemia observed in db/db is consistent with the previously described db/db mice (Burke et al., 2017) and patients with T2D (Sung et al., 2011) and represents the first report in mice susceptible to malignant hyperthermia. It is worth noting that insulin resistance and hyperinsulinemia are often associated with the development of a specific form of cardiomyopathy, diabetic cardiomyopathy, a condition in which heart failure occurs in the absence of coronary artery disease and represents the leading cause of death in patients with T2D (Dillmann, 2019; Uryash et al., 2021). In the light of our previous and present observations, it is reasonable to conclude that a chronic increase in muscle [Ca^2+^]_i_ diminishes muscle responsiveness to insulin-glucose uptake and subsequently provokes muscle glucose intolerance.

### Effects of Dantrolene on [Ca^2+^]_i,_ Glucose Uptake and Hyperglycemia

We tested among the numerous potential abnormalities that account for or contribute to muscle insulin resistance, the possibility that sustained elevated levels [Ca^2+^]_i_ could make these cells resistant to insulin, a specific feature of T2D (DeFronzo and Tripathy, 2009). Dantrolene treatment in *RYR1*-p.163C and db/db mice causes a significant reduction in fasting blood glucose levels and reduces the maximum amplitude after glucose load in *RYR1*-p.R163C and db/db mice. Additionally, at the cellular level, dantrolene significantly reduced muscle [Ca^2+^]_i_ in all three models but improved insulin-induced glucose uptake (*in vivo* and *in vitro*) only in *RYR1*-p.R163C and db/db muscle cells. The effects of dantrolene on [Ca^2+^]_i_ have been described previously in muscle cells from humans (Lopez et al., 1992), swine (Lopez et al., 1987a), and mice (Yang et al., 2006; Cherednichenko et al., 2008; Eltit et al., 2013). The mechanism by which dantrolene reduced [Ca^2+^]_i_ in skeletal muscle is unclear. Predominant facts support the view that dantrolene effects appear to be mediated primarily on the RyR1 receptor, inhibiting Ca^2+^ release from the sarcoplasmic reticulum (Helland et al., 1988; Fruen et al., 1997). However, other mechanisms have been proposed for the pharmacological effects of dantrolene on skeletal muscle, such as reducing Ca^2+^ leakage from the RyR1 receptor (Yang et al., 2007) and Ca^2+^ entry into muscle cells (Cherednichenko et al., 2008). Therefore, dantrolene may have more than one mechanism to modify [Ca^2+^]_i_ in skeletal muscle.

Our data show that a significant reduction in [Ca^2+^]_i_ induced by dantrolene (20 nM) in the *RYR*1-p.R163C and db/db muscles transformed them from insulin resistance muscle to noninsulin resistance muscle (glucose tolerance muscle). This finding supports the hypothesis of the existence of a [Ca^2+^]_i_ window for muscle glucose transport, with an optimal [Ca^2+^]_i_ in the range of 90–130 nM (Figures 5A,D) while reduction of [Ca^2+^]_i_ outside of this “ideal window” (<90 nM) or elevation (>150 nM) causes markedly inhibition of insulin-stimulated glucose transport. Therefore, [Ca^2+^]_i_ modulates insulin-mediated glucose transport in skeletal muscle in two opposite ways: a permissive effect as long as [Ca^2+^]_i_ remains at optimal concentration and an inhibitory effect, caused by chronic reduction or elevation of muscle [Ca^2+^]_i_, which inhibits insulin-mediated glucose uptake causing muscle insulin resistance and hyperinsulinemia, as observed in *RYR1*-p.R163C and db/db mice.

### GLUT-4 Glucose Transporter

Our western blot studies demonstrated that overall GLUT4 expression was significantly lower in *RYR1*-pR163C and db/db muscle compared to WT. Our finding coincides with Park et al. (2009), who demonstrated that muscle GLUT4 expression is inhibited by chronic elevation of [Ca^2+^]_i_ induced by the high concentration or prolonged exposure to caffeine. A direct relationship between muscle GLUT4 content and glucose uptake in response to insulin stimulation has been described in muscle cells (Henriksen et al., 1990); therefore, the reduction in GLUT4 expression may explain the abnormal glucose uptake observed in the *RYR1*-pR163C and db/db muscle.

We observed an interesting difference in the fractionation distribution of the GLUT4 subcellular protein between *RYR1*-pR163C and the db/db and WT muscles. The fraction of GLUT4 subcellular membrane was reduced in *RYR1*-pR163C and db/db muscle compared to WT. On the contrary, the cytoplasmic fraction of GLUT4 was more elevated in the *RYR1*-pR163C and db/db muscles than in WT. The translocation of GLUT4 to the plasma membrane in skeletal muscle after insulin stimulation represents the combination of two intricate systems: *i)* a signal transduction and *ii)* a vesicular transport (Stockli et al., 2011). Under basal conditions, GLUT4 glucose transporters are stored in cytoplasmatic insulin-responsive vesicles, and upon binding of insulin to the insulin receptor, GLUT4 is translocated to the cell surface to facilitate glucose transport. A transient elevation of intracellular Ca^2+^ appears to be essential for the recruitment of GLUT4 to the plasma membrane and T tubule and the subsequent increase in glucose uptake (Mitani et al., 1996; Jensen et al., 2007). However, the link between the insulin-signaling cascade and the events connected with GLUT4 trafficking are not fully understood. Based on the present results, it is appealing to suggest that the difference in the expression and fractionation distribution of GLUT4 observed in the *RYR1*-pR163C and db/db muscles could also be associated with elevated [Ca^2+^]_i_. In this regard, chronic elevation [Ca^2+^]_i_ caused phosphorylation and reduced GLUT4 intrinsic activity in adipocytes, and treatment with nitrendipine, a Ca^2+^ blocker, improved the ability of insulin to dephosphorylate GLUT4 and restored insulin-stimulated GLUT4 (Begum et al., 1993; Reusch et al., 1993).

### Study Limitations

Despite the impact on the present finding, some limitations should be acknowledged. Muscle resistance to insulin is aggravated with aging, regardless of its association with insufficient insulin secretion. In the current study, we used only 3-4-month-old *RYR1*-pR163C and db/db mice, not aged mice. We did not explore the dose-response effect of dantrolene on muscle GLUT4 expression and its subcellular fractionation in *RYR1*-pR163C and db/db muscle. Furthermore, we did not study the mechanism by which [Ca^2+^]_i_ modulates the global expression and translocation of GLUT4 and the degree of GLUT4 phosphorylation in the *RYR1*-pR163C and db/db muscle.

## Conclusion

Collectively, this novel study provides information on the unexplored crosstalk between [Ca^2+^]_i_ and glucose uptake in skeletal muscle. Using two experimental models, *RYR1*-p.R163C and db/db mice, we have shown *in vivo* and *in vitro* that chronic elevation of [Ca^2+^]_i_ impair insulin-stimulated muscle glucose uptake, causing whole-body glucose dyshomeostasis. The Ca^2+^-induced glucose uptake impairment appears to reside in part in the abnormal GLUT4 expression and its subcellular distribution. Consistent with our hypothesis, reducing [Ca^2+^]_i_ improves glucose uptake and whole-body glucose homeostasis. Furthermore, this study aims to use skeletal muscle as a target to improve glucose dyshomeostasis in patients with T2D, a therapeutic approach that has been overlooked.

## Data Availability

The raw data supporting the conclusion of this article will be made available by the authors, without undue reservation.

## Abbreviations

AUC, area under the curve; DANT, dantrolene; GLU, glucose; GTT, glucose tolerance test; MH, malignant hyperthermia; millisecond, msec; T tubules, transverse tubules; T2D, type 2 diabetes; [Ca^2+^]_i_, resting intracellular calcium concentration.
